# Clinicopathological and Functional Evaluation Reveal NBS1 as a Predictor of Platinum Resistance in Epithelial Ovarian Cancers

**DOI:** 10.3390/biomedicines9010056

**Published:** 2021-01-08

**Authors:** Adel Alblihy, Muslim L. Alabdullah, Reem Ali, Mashael Algethami, Michael S. Toss, Nigel P. Mongan, Emad A. Rakha, Srinivasan Madhusudan

**Affiliations:** 1 Translational Oncology, Division of Cancer and Stem Cells, School of Medicine, University of Nottingham Biodiscovery Institute, Nottingham NG5 1PB, UK; msxamal@exmail.nottingham.ac.uk (A.A.); msxmla@exmail.nottingham.ac.uk (M.L.A.); drreemali7@gmail.com (R.A.); msxma58@exmail.nottingham.ac.uk (M.A.); 2 Medical Center, King Fahad Security College (KFSC), Riyadh 11461, Saudi Arabia; 3 Academic Pathology, Division of Cancer and Stem Cells, School of Medicine, University of Nottingham Biodiscovery Institute, Nottingham NG5 1PB, UK; mszmst@exmail.nottingham.ac.uk (M.S.T.); mrzear1@exmail.nottingham.ac.uk (E.A.R.); 4 School Veterinary Medicine and Science, Faculty of Medicine and Health Sciences, University of Nottingham Biodiscovery Institute, Nottingham NG7 2RD, UK; svznpm@exmail.nottingham.ac.uk; 5 Department of Pharmacology, Weill Cornell Medicine, New York, NY 10065, USA; 6 Department of Oncology, Nottingham University Hospitals, Nottingham NG5 1PB, UK

**Keywords:** NBS1, ovarian cancer, platinum sensitization, biomarker

## Abstract

Platinum resistance seriously impacts on the survival outcomes of patients with ovarian cancers. Platinum-induced DNA damage is processed through DNA repair. NBS1 is a key DNA repair protein. Here, we evaluated the role of NBS1 in ovarian cancers. NBS1 expression was investigated in clinical cohorts (protein level (*n* = 331) and at the transcriptomic level (*n* = 1259)). Pre-clinically, sub-cellular localization of NBS1 at baseline and following cisplatin therapy was tested in platinum resistant (A2780cis, PEO4) and sensitive (A2780, PEO1) ovarian cancer cells. NBS1 was depleted and cisplatin sensitivity was investigated in A2780cis and PEO4 cells. Nuclear NBS1 overexpression was associated with platinum resistance (*p* = 0.0001). In univariate and multivariate analysis, nuclear NBS1 overexpression was associated with progression free survival (PFS) (*p*-values = 0.003 and 0.017, respectively) and overall survival (OS) (*p*-values = 0.035 and 0.009, respectively). NBS1 mRNA overexpression was linked with poor PFS (*p* = 0.011). Pre-clinically, following cisplatin treatment, we observed nuclear localization of NBS1 in A2780cis and PEO4 compared to A2780 and PEO1 cells. NBS1 depletion increased cisplatin cytotoxicity, which was associated with accumulation of double strand breaks (DSBs), S-phase cell cycle arrest, and increased apoptosis. NBS1 is a predictor of platinum sensitivity and could aid stratification of ovarian cancer therapy.

## 1. Introduction

Overall survival outcomes for patients with advanced ovarian cancer remains poor despite platinum-based chemotherapy [1,2,3,4]. The development of platinum resistance and disease recurrence is a formidable clinical problem [5,6,7]. Although the mechanism of action of platinating agents (carboplatin, cisplatin) is complex, the cytotoxicity predominantly is related to their ability to induce intra-strand or inter-strand DNA cross-links in cancer cells [8]. If unrepaired, the DNA cross-links can get converted to double strand breaks (DSBs) during replication. Accumulation of DSBs can promote cancer cell death [8]. However, enhanced DNA repair capacity and processing of DSBs can lead to platinum resistance in cancer [9]. Recently, in platinum sensitive sporadic or BRCA germ-line deficient ovarian cancers, PARP inhibitor (Olaparib, Niraparib, Rucaparib, Talazoparib) maintenance therapy was shown to improve survival [10]. However, only 50% of patient with platinum sensitive disease obtain benefit from this approach [11]. Therefore, the search for biomarker(s), besides *BRCA* mutation status, that determine platinum sensitivity is highly desirable in ovarian cancers.

The MRE11-RAD50-NBS1 (MRN) complex is key sensor of DSBs [12,13,14,15,16,17]. The NBS1 protein (also referred to as NBN, nibrin) is a key component of the MRN complex [17,18,19,20]. NBS1 modulates DNA damage response (DDR) by recruiting and activating ATM and ATR to sites of DNA damage. In addition, NBS1 is also involved in the control of intra-S-phase checkpoints through the activation of ATM and CHK2 after ionizing radiation (IR). *NBS1* null mutations in mice are embryonic lethal. Germ-line mutation in the *NBS1* gene causes Nijmegen breakage syndrome (NBS), a rare autosomal recessive disorder characterized by cancer predisposition, microcephaly, growth retardation, immunodeficiency, and radio sensitivity. *NBS1* mutations and polymorphisms can increase risk of many cancers including ovarian cancers [17,18,19,20,21]. We hypothesized a role for NBS1 in ovarian cancer pathogenesis and response of platinum therapy.

## 2. Experimental Section

### 2.1. Clinical Study

#### 2.1.1. NBS1 Protein Expression in Ovarian Cancers

The expression of NBS1 was evaluated on tissue microarrays of 331 consecutive epithelial ovarian cancers treated at Nottingham University Hospitals (NUH) between 1997 and 2010. This study was carried out in accordance with the declaration of The Helsinki and ethical approval which was obtained from the Nottingham Research Ethics Committee (REC Approval Number 06/Q240/153). Patients were comprehensively staged as per the International Federation of Obstetricians and Gynecologists (FIGO) Staging System for Ovarian Cancer. Overall Survival was calculated from the operation date until the 1st of October 2016, when any remaining survivors were censored. All patients received platinum based chemotherapy. Platinum resistance was defined as patients who had progression during first-line platinum chemotherapy or relapse within 6 months after completion of chemotherapy. Progression-free survival was calculated from the date of the initial surgery to disease progression or from the date of the initial surgery to the last date known to be progression-free for those censored. Patient demographics are summarized in Supplementary Table S1.

#### 2.1.2. Tissue Microarray (TMA) and Immunohistochemistry (IHC)

Tumors were arrayed in tissue microarrays (TMAs) constructed with 2 replicate 0.6 mm cores from the tumors. Immunohistochemical staining was preformed using the Thermo Fisher Scientific Shandon Sequenza chamber system (REF: 72110017, Cheshire, UK), in combination with the Novolink Max Polymer Detection System (RE7280-K: 1250 tests, Buffalo Grove, IL, USA), and the Leica Bond Primary Antibody Diluent (AR9352, Buffalo Grove, IL, USA), each used according to the manufacturer’s instructions (Leica Microsystems Buffalo Grove, IL, USA). The TMA slides were deparaffinized with xylene and then rehydrated through five decreasing concentrations of alcohol (100%, 90%, 70%, 50%, and 30%) for two minutes each. Pre-treatment antigen retrieval was carried out on the TMA sections using sodium citrate buffer (pH 6.0) and heated at 95 °C in a microwave (Whirlpool JT359 Jet Chef 1000W, UK) for 20 min. A set of slides were incubated with the primary anti-NBS1 rabbit monoclonal antibody (N3162, Sigma, Gillingham, Dorset, UK), at a dilution of 1:200, for 60 min at room temperature. Negative (by omission of the primary antibody and IgG-matched serum) and positive controls were included in each run.

#### 2.1.3. Evaluation of Immune Staining

Whole field inspection of the core was scored, and the subcellular localization of each marker was identified (nuclear, cytoplasm, cell membrane). Intensities of subcellular compartments were each evaluated and grouped as follows: 0 = no staining, 1 = weak staining, 2 = moderate staining, 3 = strong staining. The percentage of tumor cells in each category was estimated (0–100%). Histochemical score (H-score) (range 0–300) was calculated by multiplying the intensity of staining and the percentage of staining. A median H-score of ≤80 and ≤90 was used as the cut-off for high NBS1 nuclear and cytoplasmic expression, respectively. Not all cores within the TMA were included for IHC analysis due to missing cores or absence of tumor cells.

#### 2.1.4. Statistical Analysis

Correlation with clinical and pathological characteristics using categorized data was calculated using Chi-squared test. All tests were 2-tailed. Survival rates were determined using Kaplan–Meier method and compared by the log-rank test. All analyses were conducted using Statistical Package for the Social Sciences (SPSS, version 22, Chicago, IL, USA) software for windows. *p* value <0.05 was identified as statistically significant.

#### 2.1.5. NBS1 Transcript in Ovarian Cancers

*NBS1* mRNA expression was assessed in a publicly available online gene expression dataset of 1259 ovarian cancer patients treated with platinum-based chemotherapy from 15 previously published studies and available at ‘http://kmplot.com/analysis/index.php?p=service&cancer=ovar’.

### 2.2. Pre-Clinical Study

#### 2.2.1. Cell Lines and Tissue Culture

PEO1 (BRCA2-deficient) and PE04 (BRCA2-proficient) were purchased from American Type Culture Collection (ATCC, Manassas, VA, USA). A2780 (platinum sensitive) A2780cis (platinum resistant) were purchased from Sigma Aldrich (Gillingham, UK). Cells cultured in RPMI (R8758, Merck, Gillingham, Dorset, UK) supplemented with 1% Penicillin-Streptomycin (P4333, Merck Gillingham, Dorset, UK) and 10% FBS (F4135, Merck Gillingham, Dorset, UK). All cell lines were maintained in a humidified incubator at 37 °C in a 5% CO_2_ atmosphere.

#### 2.2.2. Nuclear/Cytoplasmic Extracts and Western Blot Analysis

Cells were collected by trypsinization, washed with PBS, and centrifuged at 1000×g for 5 min. The extraction of nuclear and cytoplasmic lysates were preformed using the NE-PER Nuclear and Cytoplasmic Extraction Reagents (78833, Thermo Fisher, Cheshire, UK). Extracts were quantified using BCA protein quantification kit and protein levels were checked by western blot. Samples were run on SDS-bolt gel (4–12%) bis-tris. Membranes were then incubated with primary antibodies as follows: NBS1 (1:500, N3162, sigma, Gillingham, Dorset, UK), YY1 (1:1000, ab109228), ß-actin (1:1000, ab8226), GADPH (1:1000, ab9485). Membranes were then washed and incubated with Infrared dye-labelled secondary antibodies (LiCor) (IRDye 800CW Donkey Anti-Rabbit IgG (926-32213) and IRDye 680CW Donkey Anti-Mouse IgG (926-68072)) at dilution of 1:10,000 for 60 min. Membranes were scanned with a LiCor Odyssey machine (700 and 800 nm, Cambridge, UK) to determine protein levels.

#### 2.2.3. Protein Quantification

The Pierce BCA kit assay by Thermo Fisher Scientific was used for protein quantification. The standard curve was performed with BSA (working range 25 to 2000 μg/mL) following manufacturer’s instructions. Samples were then added to a 96-well micro plate and 200 μL of working reagent (50 parts of BCA Reagent A with 1 part of BCA Reagent B) was added either to samples or standard curve. The plate was incubated at 37 °C for half an hour in the dark, and the absorbance was measured by FLUOstar OPTIMA, UK/Infinite^®^ F50 (Cheshire, UK) microplate reader at 590 nm. Standards and unknown samples were performed in duplicate.

#### 2.2.4. Transient Knockdowns of NBS1

NBS1 (ID S9292) and the validation construct of NBS1 (ID S9293) siRNAs oligonucleotides were purchased from Invitrogen. Lipofectamine 3000 reagent (L3000015, Invitrogen, Cheshire, UK) was used according to the manufacturer’s protocol. Briefly, cells were plated at 50–60% confluency in T25 flasks overnight. In the following day, cells were transfected with 20 nM of siRNA oligonucleotide or scrambled SiRNA oligonucleotide control (4390843, Thermo Fisher, Cheshire, UK) in Opti-MEM media (31985-062, Gibco, Merck, Gillingham, Dorset, UK). The efficiency of transfection was confirmed using western blot.

#### 2.2.5. Clonogenic Assays

In the clonogenic assay, 32 cells/cm^2^ were seeded in 6-well plates and left at 37 °C in a 5% CO_2_ atmosphere. Cisplatin (kindly provided by Nottingham University Hospital, Nottingham, UK) was added at the indicated concentrations and the plates were left at 37 °C in a 5% CO_2_ atmosphere for two weeks. The plates were then washed with PBS, fixed and stained, and colonies were counted.

#### 2.2.6. DSB Accumulation, Cell Cycle and Apoptosis Analysis by Flow Cytometry

1 × 10^5^ cells per well were seeded in 6-well plates overnight. Cells were treated with cisplatin (1 μM) for A2780 cells and (5 μM) for A2780 cis cells. After 24 h, cells were trypsinized and washed with ice cold PBS and then fixed in 70% ethanol for at least 30 min. After removal of the fixative solution by centrifugation cells were stained with anti-phospho Histone (γH2AX) Ser139. Cells were then treated with RNase and DNA content were stained with 10 ug/mL propidium iodide (Sigma Aldrich) in PBS. For apoptosis detection, cells were collected by trypsinization after 24 h washed and analyzed using annexinV detection kit (BD biosciences). Samples were analyzed on FC500 flow cytometer (Beckman Coulter, High Wycombe, UK), and data were analyzed using Weasel software (version 3.7.1., Helsinki, Finland).

#### 2.2.7. Statistical Analysis

Data analysis was conducted on GraphPad Prism 7 software (version 5,). To compare between two groups, Student’s T-tests analysis was performed. One-way ANOVA was performed to compare between more than two groups (variances analyses). Two-way ANOVA was used to analyze two variables, such as Annexin V analysis and cell cycle analysis. All experiments were expressed as means ± standard deviation S.D. of three independent experiments. The *p*-values < 0.05 = *, *p*-value < 0.01 = ** & *p*-value < 0.001 = ***.

#### 2.2.8. Next Generation Sequencing and Bioinformatics

Genomic DNA was extracted from cell lines using the PicoPure™ DNA Extraction Kit (Thermo Fisher, Cheshire, UK). Next generation sequencing was used to identify genomic variants in platinum sensitive (A2780) and platinum resistant derivatives (A2780cis). The SureSelect All Exon V5 kit (Agilent Technologies, Santa Clara, CA, USA) was used to enrich for protein coding regions and sequencing performed using an Illumina NextSeq500 sequencer with paired end reads (150 bp) and a minimum of 80 million reads generated per sample. Contaminating adapter sequences and low-quality sequences were processed using Skewer [22]. Quality processed reads were aligned to the HG19 reference genome using BWA [23], duplicate alignments identified and processed using PicardTools, and realignment completed using the Abra assembly based realigner [24] to enhance detection of insertion/deletion variants. Variant calling and filtering was completed using Samtools/Bcftools (Version 1.3.1) [25]. Variants, in variant call format (VCF), associated with platinum resistance were identified using Vcftools [26]. Variants were annotated and functional significance assessed using the Ensembl Variant Effect Predictor tool [27]. Library preparation and sequencing was conducted by Source Biosciences (Nottingham, UK).

## 3. Results

### 3.1. NBS1 Overexpression and Platinum Resistant Aggressive Ovarian Cancers

**Figure 1:**
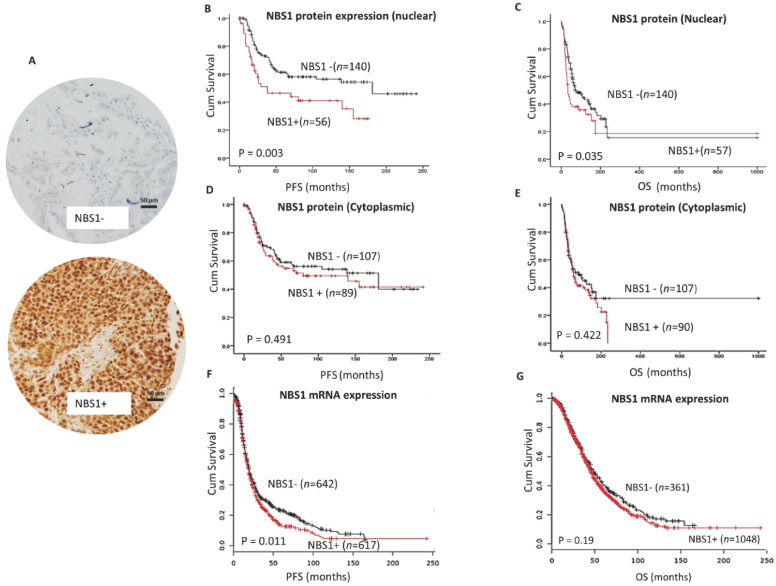
NBS1 and epithelial ovarian cancers. (**A**) Immunohistochemical staining of NBS1 in ovarian cancers. (**B**) Kaplan-Meier curve for NBS1 nuclear protein expression and progression free survival (PFS) in ovarian cancer. (**C**) Kaplan-Meier curve for NBS1 nuclear protein expression and overall survival (OS) in ovarian cancer. (**D**) Kaplan-Meier curve for NBS1 cytoplasmic protein expression and PFS in ovarian cancer. (**E**) Kaplan-Meier curve for NBS1 cytoplasmic protein expression and OS in ovarian cancer. (**F**) Kaplan-Meier curve for NBS1 mRNA expression and PFS in ovarian cancer. (**G**) Kaplan-Meier curve for *NBS1* mRNA expression and OS in ovarian cancer. Scale bar = 50 µM, red line= high expression, black line = low expression.

We investigated the clinicopathological significance of NBS1 protein expression by immunohistochemistry (Figure 1A) in a clinical cohort of human ovarian cancers. Patient demographics are summarized in Supplementary Table S1. NBS1 protein expression was evaluable in 225 tumors. NBS1 nuclear overexpression was seen in 56/225 (28.5%) tumors and was significantly associated with platinum resistance (*p* = 0.0001) (Table 1), shorter progression free survival (PFS) (*p* = 0.003) (Figure 1B), and poor OS (*p* = 0.035) (Figure 1C). High cytoplasmic NBS1 expression was linked to serous cystadenocarcinoma (*p* = 0.00004) (Supplementary Table S2) but did not influence survival (Figure 1D,E). In multivariate analysis (Table 2), Nuclear NBS1 was independently associated with PFS (*p* = 0.017) and OS (*p* = 0.009).

For additional validation, we evaluated clinical significance of NBS1 mRNA expression in a publicly available data set (http://kmplot.com/analysis/index.php?p=service&cancer=ovar) of ovarian cancers (*n* = 1259). At the transcriptomic level, *NBS1* mRNA overexpression was significantly associated with poor PFS (*p* = 0.011) (Figure 1F) but did not influence on OS (*p* = 0.11) (Figure 1G).

Taken together, the data suggest that NBS1 could be a predictor of platinum resistance and poor clinical outcome in patients. We proceeded to pre-clinical functional investigations.

### 3.2. Sub-Cellular Localization of NBS1 in Ovarian Cancer Cells Following Cisplatin Therapy

**Figure 2:**
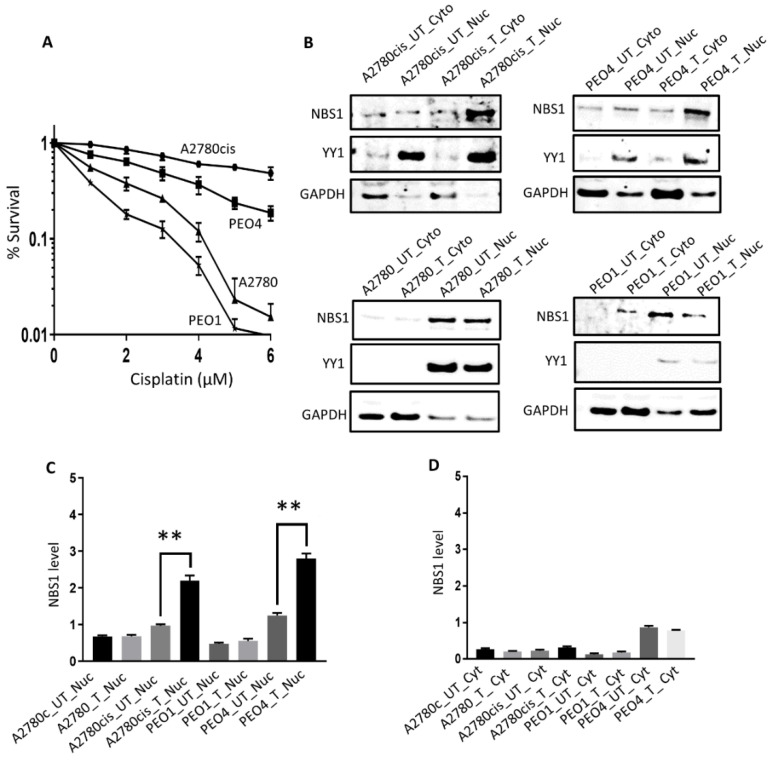
Sub-cellular localization of NBS1 following cisplatin in ovarian cancer cells. (**A**) Clonogenic assay showing Cisplatin sensitivity in A2780, PEO1 compared to A2780cis and PEO4 cells. (**B**) NBS1 level nuclear and cytoplasmic extracts in platinum resistant (A2780cis, PEO4) and platinum sensitive (A2780, PEO1) cells treated with 5µM cisplatin. Lysates collected 48 h post-treatment. (**C**) NBS1 nuclear level quantification A2780, A2780cis, PEO1, and PEO4 cells. (**D**) NBS1 cytoplasmic level quantification A2780, A2780cis, PEO1, and PEO4 cells. UN = untreated, T = treated. ‘**’ = *p* value < 0.001.

A2780 cell is platinum sensitive cell line which was established from previously untreated ovarian cancer patient. A2780cis cell is a platinum resistant cell line developed by continuous exposure of the A2780 cell to increasing doses of cisplatin. PEO1 is a platinum sensitive (BRCA2-deficient) cell line which was derived from a poorly differentiated serous adenocarcinoma patient who was treated with platinum agents. PEO4 platinum resistant (BRCA2-proficient) cell line was derived from a malignant effusion from the peritoneal ascites of the same patient after the development of clinical resistance to platinum therapy. We first confirmed platinum sensitivity (A2780, PEO1) and resistance (A2780cis, PEO4) by clonogenic assays (Figure 2A). NBS1 protein levels in nuclear and cytoplasmic extracts at baseline and following 48 h cisplatin treatment were then investigated in A2780, A2780cis, PEO1, and PEO4 cells (Figure 2B). In platinum resistant A2780cis and PEO4 cells, platinum treatment increased nuclear sub-cellular localization of NBS1 compared to in platinum sensitive A2780 and PEO1 cells (Figure 2C). However, no significant alterations were seen for NBS1 cytoplasmic expression in A2780, A2780cis, PEO1, and PEO4 cells (Figure 2D). The results indicate that accumulation of nuclear NBS1 in A2780cis and PEO4 cells could contribute to cisplatin resistance.

### 3.3. NBS1 Variant Profiling in A2780, A2780cis, PEO1, and PEO4 Cells

As *NBS1* mutation or polymorphic variants are linked with increased cancer risk [17,18,19,20,21], we performed targeted deep sequencing for *NBS1* variants in A2780, A2780cis, PEO1 and PEO4 cells. Ensembl VEP was used to analyze the effect and location of variants using the HG19/GRCh37 genome version. The platinum resistant A2780cis cell line harbors two novel missense mutations Gly214Arg and Asn209Tyr affecting the NBS1 function (ENST00000265433.3). These changes result in a charge and volume substitutions, respectively. Mutations in adjacent amino acids have been described in melanoma, uterine and colorectal cancers. There is no crystal structure for this region of NBS1, and little functional knowledge about this domain is available. However, based on homology, we observed that this region of the protein may mediate interaction with the Sp100, a potent tumor suppressor [28]. Together, the data provides evidence of NBS1 mutant ovarian cancer cells.

We have also investigated Mre11 and Rad50 variants in ovarian cancer cells. In the parental A2780 line, two unique variants were identified (A: 5:131893147-131893147, a novel variant predicted to alter splicing; B: 5:131977963-131977963, rs1804670, a synonymous variant). The Platinum-resistant A2780cis harbors a novel unique variant at 5:131973821-131973821 which is predicted to introduce Ala→Asp amino acid substitution. This variant is located within the ATPase domain of RAD50. While the Ala→Asp substitution is similar in size and volume, the introduction of an acidic aspartic acid may influence substrate access to the ATPase domain. No variants were identified for Mre11.

### 3.4. NBS1 Depletion and Platinum Sensitivity

**Figure 3:**
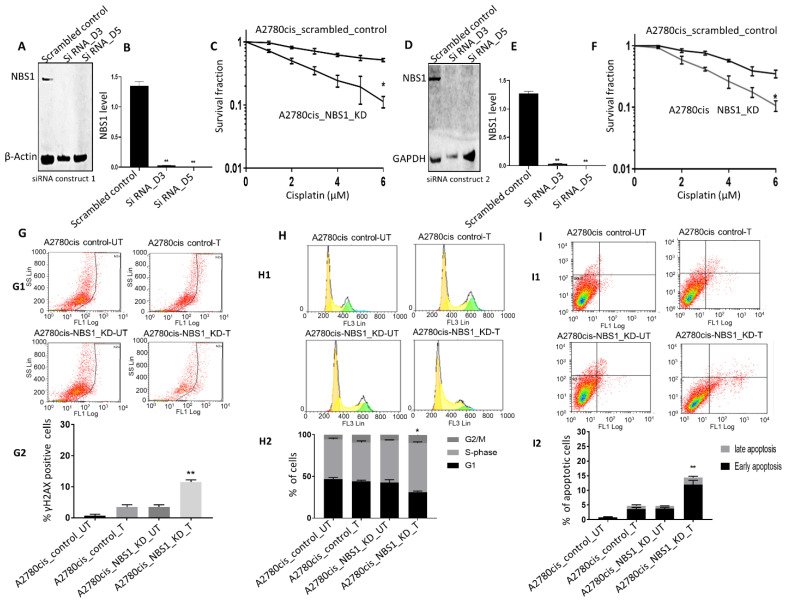
NBS1 depletion and platinum sensitivity in A2780cis cells. (**A**) NBS1_KD in A2780cis cells using siRNA construct 1. (**B**) NBS1 protein quantification in scrambled control and siRNA construct 1 in A2780cis cells. (**C**) Cisplatin sensitivity in A2780cis control and A2780cis_RAD50_KD cells using siRNA construct 1. (**D**) NBS1_KD in A2780cis cells using siRNA construct 2. (**E**) NBS1 protein quantification in scrambled control and siRNA construct 2 in A2780cis cells. (**F**) Cisplatin sensitivity in A2780cis control and A2780cis_RAD50_KD cells using siRNA construct 2. (**G**) Representative data showing percentage of γH2AX positive cells by flow cytometry in control and NBS1_KD A2780cis cells untreated or treated with cisplatin (**G1**) and quantification of % γH2Ax positive cells by flowcytometry (**G2**) (FL1 Log: FACS fluorescence channel used to measure yH2AX staining). (**H**) Representative data showing cell cycle progression by flow cytometry in control and NBS1_KD in A2780cis cells untreated or treated with cisplatin (**H1**) and quantification of cell cycle analysis by flow cytometry (**H2**) (FL3 Lin: FACS fluorescence channel used for measuring PI staining). (**I**) Representative data showing apoptotic cells accumulation by flow cytometry in control and NBS1_KD in A2780cis cells untreated or treated with cisplatin (**I1**) and quantification of apoptotic cells by flow cytometry (**I2**) (FL1 Log: FACS fluorescence channel used to measure Annexin V staining (horizontal) and FL3 log to measure PI staining (vertical). Early apoptosis rate is calculated from FL1+/PI−, while late apoptosis rate is from FL1+/PI+). UN = untreated, T = treated. ‘*’ = *p* < 0.01, ‘**’ = *p* < 0.001.

**Figure 4:**
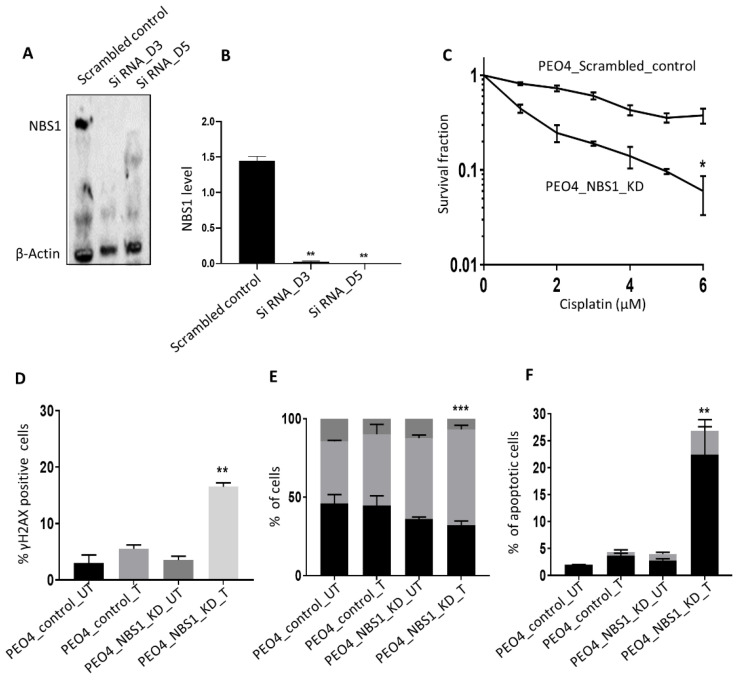
(**A**) NBS1_KD in PEO4. (**B**) NBS1 protein quantification in scrambled control and siRNA construct 1 in PEO4 cells. (**C**) Cisplatin sensitivity in PEO4 control and PEO4_NBS1_KD cells. (**D**) The percentage of γH2Ax positive cells by flowcytometry. (**E**) Cell cycle analysis by flow cytometry (**F**) annexin V apoptosis analysis by flow cytometry. UN = untreated, T = treated. ‘**’ = *p* < 0.001, ‘***’ = *p* < 0.0001.

To evaluate the predictive significance of NBS1, we then proceeded to generate transient knockdowns (KD) of NBS1 using siRNA constructs in platinum resistant A2780cis cells (Figure 3A,B). In clonogenic assays, NBS1_KD_A2780cis cells were significantly sensitive to cisplatin compared to scrambled control (Figure 3C). We also confirmed platinum sensitization using second siRNA construct to deplete NBS1 in A2780 cis cells (Figure 3D–F) compared to scrambled controls. Increased platinum cytotoxicity in NBS1 depleted cells was associated with DSB accumulation (Figure 3G), S-phase cell cycle arrest (Figure 3H), and increased apoptosis (Figure 3I) compared to scrambled controls. For further validation, we depleted NBS1 in PEO4 cells using siRNA (Figure 4A,B). In clonogenic assays, as expected, NBS1_KD_PEO4 cells (Figure 4C) were sensitive to cisplatin treatment compared to controls. Increased sensitivity was associated with DSB accumulation (Figure 4D), S-Phase arrest (Figure 4E), and increased apoptotic cells (Figure 4F). Taken together, the clinical and pre-clinical data provides evidence that NBS1 is a predictor of platinum sensitivity in epithelial ovarian cancers.

## 4. Discussion

Platinum resistance is a clinical challenge during ovarian cancer therapy [6]. The MRE11-RAD50-NBS1 (MRN) complex is the first responder to DSBs [12,13,14]. NBS1 has a vital role in repairing DSBs via non-homologous and joining (NHEJ) and homologous recombination (HR) [17,18,19,20]. Clinical and pre-clinical evidence presented here provides evidence that NBS1 is a predictor of platinum sensitivity in ovarian cancers.

In clinical cohort of ovarian cancer, we observed that high nuclear expression was associated with platinum resistance and poor PFS in patients. Our data concurs with a previous study in Asian ovarian cancer patients, where NBS1 overexpression was shown to be associated with aggressive phenotype and poor survival [29]. Although this study did not report separate nuclear and cytoplasmic expression, overall NBS1 expression was associated with advanced stage, serous histology, high grade, and residual tumor in that study [29]. In the current study, only cytoplasmic overexpression of NB1 was associated with serous cancers. Interestingly, another study has demonstrated that low NBS1 expression was observed in low-grade ovarian tumors [30]. NBS1 overexpression was also linked to aggressive phenotypes in oral squamous cell carcinoma (OSCC) [31], head and neck cancer [32], uveal melanoma [33], gastric cancer [34], and colorectal cancer [35]. However, all these studies including ours are retrospective, which is a limitation. Although prospective studies will be required, taken together, the data provides clinical evidence that NBS1 is a promising predictive biomarker in solid tumors. Another limitation of our study is that we have not DNA sequenced individual tumors to identify any genetic variants of *Mre11, Rad50*, nor *NBS1* genes.

In pre-clinical studies, we observed that depletion of NBS1 increased platinum sensitivity. This supports a model for platinum sensitivity were a reduction in NBS1 levels disrupts the stability of MRN complex, thereby impairing DNA damage recognition and repair. Persistent cisplatin induced DNA intra-strand and inter-strand cross link during replication will result in replication arrest, generation and accumulation of DSBs, and apoptosis. Consistent with this model, we observed DSB accumulation, S-phase arrest and increased apoptotic cells in NBS1 depleted cells compared to controls. A limitation to our study is that we investigated transient knock-down of NBS1 using siRNA. Evaluation in stable KD (using shRNAs) or CRISPR knock out system and in vivo studies will be required to validate our findings in ovarian cancers. Interestingly, in head and neck cancer models, molecular disruption of NBS1 increased cisplatin sensitivity in xenograft models, providing in vivo evidence that NBS1 is a predictive biomarker in solid tumors [36,37]. A further limitation to the pre-clinical study is that we did not directly monitor DDR functional status in NBS1 deficient cells using assays, such as genomic scar assays [38]. However, as a surrogate marker of DSB accumulation, we quantified the percentage of γH2AX positive cells as a marker of DNA damage by flow cytometry [39] in control and NBS1 deficient cells before and after cisplatin treatment.

In platinum sensitive sporadic ovarian cancers, PARP inhibitor maintenance therapy significantly improves PFS in patients [10]. Although BRCA germ line mutation is a strong predictor of platinum sensitivity and benefits from PARP inhibitors maintenance therapy, biomarkers of benefit in sporadic ovarian cancers are yet to be defined. In the current study, we have shown that NBS1 deficiency is a predictor of platinum sensitivity. McCabe et al., previously, have shown that NBS1 deficient human SV40- transformed immortal fibroblasts were sensitive to PARP inhibitor treatment compared to isogenic NBS1 proficient fibroblasts [40]. In NBS1, deficient breast cancer cell line models were also sensitive to PARP inhibitors in another study [41]. Taken together, the data would suggest that NBS1 depletion is not only a predictor of platinum sensitivity but could also aid in defining those patients who could benefit from PARP inhibitor maintenance therapy.

## Figures and Tables

**Table 1:** NB1 protein expression and platinum resistance in sporadic ovarian cancer.

**NBS Protein Expression (Nuclear)**			***p*-Value**
	**Low**	**High**	0.0001
Sensitive	131 (74.9%)	44 (25.1%)	
Resistant *	5 (31.3%)	11 (68.8%)	
**NBS Protein Expression (Cytoplasmic)**			***p*-Value**
	**Low**	**High**	0.676
Sensitive	97 (55.4%)	78 (44.6%)	
Resistant *	8 (50%)	8 (50%)	

* = Platinum resistance was defined as patients who had progression during first-line platinum chemotherapy or relapse within 6 months after completion of chemotherapy.

**Table 2:** Multivariate analysis.

**Progression Free Survival (PFS)**				
	**Sig.**	**Exp(B)**	**95.0% CI for Exp(B)**	
			Lower	Upper
NBS1 (Nuclear)	0.017	1.803	1.111	2.925
NBS1 (cytoplasmic)	0.476	1.187	0.741	1.903
Tumor Stage	0.000	2.296	1.762	2.992
**Overall Survival (OS)**				
	**Sig.**	**Exp(B)**	**95.0% CI for Exp(B)**	
			Lower	Upper
NBS1 (nuclear)	0.009	1.746	1.147	2.658
NBS1 (cytoplasmic)	0.612	0.902	0.605	1.344
Tumor Stage	0.000	2.160	1.727	2.700

## Data Availability

Raw data can be made available upon reasonable request.

## Supplementary Materials

The following are available online at https://www.mdpi.com/2227-9059/9/1/56/s1. Table S1: Patient demographics and pathological features in ovarian cancer; Table S2: NBS1 and ovarian cancers.

## References

[1] Raja F.A., Chopra N., Ledermann J.A. (2012). Optimal first-line treatment in ovarian cancer. Ann. Oncol..

[2] Armbruster S., Coleman R.L., Rauh-Hain J.A. (2018). Management and Treatment of Recurrent Epithelial Ovarian Cancer. Hematol. Oncol. Clin. N. Am..

[3] Guan L.Y., Lu Y. (2018). New developments in molecular targeted therapy of ovarian cancer. Discov. Med..

[4] Orr B., Edwards R.P. (2018). Diagnosis and Treatment of Ovarian Cancer. Hematol. Oncol. Clin. N. Am..

[5] van Zyl B., Tang D., Bowden N.A. (2018). Biomarkers of platinum resistance in ovarian cancer: What can we use to improve treatment. Endocr. Relat. Cancer.

[6] Pujade-Lauraine E., Banerjee S., Pignata S. (2019). Management of Platinum-Resistant, Relapsed Epithelial Ovarian Cancer and New Drug Perspectives. J. Clin. Oncol..

[7] Binju M., Padilla M.A., Singomat T., Kaur P., Suryo Rahmanto Y., Cohen P.A., Yu Y. (2019). Mechanisms underlying acquired platinum resistance in high grade serous ovarian cancer—A mini review. Biochim. Biophys. Acta Gen. Subj..

[8] Fuertes M.A., Castilla J., Alonso C., Perez J.M. (2003). Cisplatin biochemical mechanism of action: From cytotoxicity to induction of cell death through interconnections between apoptotic and necrotic pathways. Curr. Med. Chem..

[9] Damia G., Broggini M. (2019). Platinum Resistance in Ovarian Cancer: Role of DNA Repair. Cancers.

[10] Pothuri B., O’Cearbhaill R., Eskander R., Armstrong D. (2020). Frontline PARP inhibitor maintenance therapy in ovarian cancer: A Society of Gynecologic Oncology practice statement. Gynecol. Oncol..

[11] D’Andrea A.D. (2018). Mechanisms of PARP inhibitor sensitivity and resistance. DNA Repair.

[12] Oh J., Symington L.S. (2018). Role of the Mre11 Complex in Preserving Genome Integrity. Genes.

[13] Paull T.T. (2018). 20 Years of Mre11 Biology: No End in Sight. Mol. Cell.

[14] Syed A., Tainer J.A. (2018). The MRE11-RAD50-NBS1 Complex Conducts the Orchestration of Damage Signaling and Outcomes to Stress in DNA Replication and Repair. Annu. Rev. Biochem..

[15] Tisi R., Vertemara J., Zampella G., Longhese M.P. (2020). Functional and structural insights into the MRX/MRN complex, a key player in recognition and repair of DNA double-strand breaks. Comput. Struct. Biotechnol. J..

[16] Bian L., Meng Y., Zhang M., Li D. (2019). MRE11-RAD50-NBS1 complex alterations and DNA damage response: Implications for cancer treatment. Mol. Cancer.

[17] Komatsu K. (2016). NBS1 and multiple regulations of DNA damage response. J. Radiat. Res..

[18] Antoccia A., Kobayashi J., Tauchi H., Matsuura S., Komatsu K. (2006). Nijmegen breakage syndrome and functions of the responsible protein, NBS1. Genome Dyn..

[19] Difilippantonio S., Nussenzweig A. (2007). The NBS1-ATM connection revisited. Cell Cycle.

[20] Kobayashi J., Antoccia A., Tauchi H., Matsuura S., Komatsu K. (2004). NBS1 and its functional role in the DNA damage response. DNA Repair.

[21] Gao P., Ma N., Li M., Tian Q.B., Liu D.W. (2013). Functional variants in NBS1 and cancer risk: Evidence from a meta-analysis of 60 publications with 111 individual studies. Mutagenesis.

[22] Jiang H., Lei R., Ding S.W., Zhu S. (2014). Skewer: A fast and accurate adapter trimmer for next-generation sequencing paired-end reads. BMC Bioinform..

[23] Li H., Durbin R. (2010). Fast and accurate long-read alignment with Burrows-Wheeler transform. Bioinformatics.

[24] Mose L.E., Wilkerson M.D., Hayes D.N., Perou C.M., Parker J.S. (2014). ABRA: Improved coding indel detection via assembly-based realignment. Bioinformatics.

[25] Li H., Handsaker B., Wysoker A., Fennell T., Ruan J., Homer N., Marth G., Abecasis G., Durbin R., Genome Project Data Processing Subgroup (2009). The Sequence Alignment/Map format and SAMtools. Bioinformatics.

[26] Danecek P., Auton A., Abecasis G., Albers C.A., Banks E., DePristo M.A., Handsaker R.E., Lunter G., Marth G.T., Sherry S.T. (2011). The variant call format and VCFtools. Bioinformatics.

[27] McLaren W., Gil L., Hunt S.E., Riat H.S., Ritchie G.R., Thormann A., Flicek P., Cunningham F. (2016). The Ensembl Variant Effect Predictor. Genome Biol..

[28] Negorev D.G., Vladimirova O.V., Kossenkov A.V., Nikonova E.V., Demarest R.M., Capobianco A.J., Showe M.K., Rauscher F.J., Showe L.C., Maul G.G. (2010). Sp100 as a potent tumor suppressor: Accelerated senescence and rapid malignant transformation of human fibroblasts through modulation of an embryonic stem cell program. Cancer Res..

[29] Lee Y.K., Park N.H., Lee H. (2015). Clinicopathological values of NBS1 and DNA damage response genes in epithelial ovarian cancers. Exp. Mol. Med..

[30] Brandt S., Samartzis E.P., Zimmermann A.K., Fink D., Moch H., Noske A., Dedes K.J. (2017). Lack of MRE11-RAD50-NBS1 (MRN) complex detection occurs frequently in low-grade epithelial ovarian cancer. BMC Cancer.

[31] Hsu D.S., Chang S.Y., Liu C.J., Tzeng C.H., Wu K.J., Kao J.Y., Yang M.H. (2010). Identification of increased NBS1 expression as a prognostic marker of squamous cell carcinoma of the oral cavity. Cancer Sci..

[32] Yang M.H., Chiang W.C., Chou T.Y., Chang S.Y., Chen P.M., Teng S.C., Wu K.J. (2006). Increased NBS1 expression is a marker of aggressive head and neck cancer and overexpression of NBS1 contributes to transformation. Clin. Cancer Res..

[33] Ehlers J.P., Harbour J.W. (2005). NBS1 expression as a prognostic marker in uveal melanoma. Clin. Cancer Res..

[34] Altan B., Yokobori T., Ide M., Bai T., Yanoma T., Kimura A., Kogure N., Suzuki M., Bao P., Mochiki E. (2016). High Expression of MRE11-RAD50-NBS1 Is Associated with Poor Prognosis and Chemoresistance in Gastric Cancer. Anticancer Res..

[35] Situ Y., Chung L., Lee C.S., Ho V. (2019). MRN (MRE11-RAD50-NBS1) Complex in Human Cancer and Prognostic Implications in Colorectal Cancer. Int. J. Mol. Sci..

[36] Araki K., Yamashita T., Reddy N., Wang H., Abuzeid W.M., Khan K., O’Malley B.W., Li D. (2010). Molecular disruption of NBS1 with targeted gene delivery enhances chemosensitisation in head and neck cancer. Br. J. Cancer.

[37] Tran H.M., Shi G., Li G., Carney J.P., O’Malley B., Li D. (2004). Mutant Nbs1 enhances cisplatin-induced DNA damage and cytotoxicity in head and neck cancer. Otolaryngol. Head Neck Surg..

[38] Hoppe M.M., Sundar R., Tan D.S.P., Jeyasekharan A.D. (2018). Biomarkers for Homologous Recombination Deficiency in Cancer. J. Natl. Cancer Inst..

[39] Barroso S.I., Aguilera A. (2020). Detection of DNA Double-Strand Breaks by gamma-H2AX Immunodetection. Homologous Recombination.

[40] McCabe N., Turner N.C., Lord C.J., Kluzek K., Bialkowska A., Swift S., Giavara S., O’Connor M.J., Tutt A.N., Zdzienicka M.Z. (2006). Deficiency in the repair of DNA damage by homologous recombination and sensitivity to poly(ADP-ribose) polymerase inhibition. Cancer Res..

[41] Daemen A., Wolf D.M., Korkola J.E., Griffith O.L., Frankum J.R., Brough R., Jakkula L.R., Wang N.J., Natrajan R., Reis-Filho J.S. (2012). Cross-platform pathway-based analysis identifies markers of response to the PARP inhibitor olaparib. Breast Cancer Res. Treat..

