# Supplementary material for: Clinicopathological and Functional Evaluation Reveal NBS1 as a Predictor of Platinum Resistance in Epithelial Ovarian Cancers
Source: Biomedicines. 2021 Jan 8;9(1):56. doi: 10.3390/biomedicines9010056 (PMC7826685; doi:10.3390/biomedicines9010056)
Supplement: Supplementary file 1 [file biomedicines-09-00056-s001.pdf]

Supplementary Table S1: Patient demographics and pathological features in ovarian cancer.

Characteristics		Number	Percentages
Pathology			
	Serous cystadenocarcinoma	178	53.9%
	Endometrioid	44	13.2%
	Clear cell carcinoma	24	7.2%
	Mucinous cystadenocarcinoma	46	13.8%
	Others	15	4.5%
	Mixed	17	5.1%
	Missing	7	2.3%
Grade			
	1	46	13.8%
	2	60	18%
	3	171	51.5%
	Missing	54	16.7%
Residual tumour			
	None/Microscopic	205	61.7%
	<1cm	34	10.2%
	>1-2 cm	15	4.5%
	>2cm	40	12%
	Missing	37	11.6%
FIGO Stage			
	I	123	37%
	II	49	14.7%
	III	128	38.5%
	IV	11	3.3%
	Missing	20	6.5%
Chemotherapy			
	Carboplatin monotherapy	81	24.3%
	Carboplatin + Paclitaxel	86	25.9%
	Other regimens *	26	8%
	No chemo	23	7%
	Unknown	27	22%
Platinum sensitivity			
	Sensitive	250	75.3%
	Resistant	26	7.8%
	Unknown	55	16.5%
Relapse status			
	Progression-free	169	50.9%
	Progressed/relapsed	118	35.5%
	Unknown	44	13.2%

* Other Regimens included: 3= CAP (Cyclophosphamide, Adriamycin and Cisplatin) 4 = ICON5 Trial 5 = SOCTROC Trial 6 = Carboplatin and Endoxan. 7 = Chlorambucil. For the unknown category, the majority received platinum-based chemo but because the exact regimen was not mentioned clearly in the database, they were considered as unknown. However, for these cases the platinum sensitivity status were clearly mentioned / recorded.

Supplementary Table S2. NBS1 and ovarian cancers

	**NBS1 _cyto -**	**NBS1_cyto +**	**P- value**	**NBS1_nuclear -**	**NBS1 _nuclear +**	**P- value**
Pathological Type			***0.000048***			0.27
Serous adenocarcinoma	58 (42.6%)	78 (57.4%)		92 (67.6%)	44 (32.4%)	
Mucinous adenocarcinoma	24 (88.9%)	3 (11.1%)		23 (85.2%)	4 (14.8%)	
Endometrioid carcinoma	16 (57.1%)	12 (42.9%)		22 (78.6%)	6 (21.4%)	
Clear cell carcinoma	13 (81.3%)	3 (18.8%)		11 (68.8%)	5 (31.3%)	
Mixed	4 (40%)	6 (60%)		8 (80%)	2 (20%)	
FIGO Stage			0.528			0.312
I	49 (57.6%)	36 (42.4%)		64 (75.3%)	21 (24.7%)	
II	15 (42.9%)	20 (57.1)		21 (60%)	14 (40%)	
III	49 (53.3%)	43 (46.7%)		65 (70.7)	27 (29.3%)	
IV	4 (57.1%)	3 (42.9%)		8 (80%)	2 (20%)	
Tumour Grade			0.148			0.374
Grade 1	13 (48.1%)	14 (51.9%)		22 (81.5%)	5 (18.5%)	
Grade 2	27 (65.9%)	14 (34.1)		27 (65.9%)	14 (34.1%)	
Grade 3	61 (48.8%)	64 (51.2%)		89 (71.2%)	36 (28.8%)	
Surgical Optimal Debulking			0.427			0.539
Optimally Debulked	94 (55.6%)	75 (44.4%)		121 (71.6%)	48 (28.4%)	
Not Optimally Debulked	21 (52,5%)	19 (47.5%)		29 (72.5)	11 (27.5%)	
Residual tumour			0.759			0.525
None/Microscopic	74 (54%)	63 (46%)		102 (74.5%)	35 (25.5%)	
<1cm	17 (63%)	10 (37%)		17 (63%)	10 (37%)	
1-2 cm	6 (46.2%)	7 (53.8%)		8 (61.5%)	5 (38.5%)	
>2cm	16 (53.3%)	14 (46.7%)		22 (73.3%)	8 (26.7%)	
Measurable Disease Before ChemoTherapy			0.543			0.252
Non	77 (55%)	63 (45%)		102 (72.9%)	38 (27.1%)	
Measurable	35 (54.7%)	29 (45.3%)		43 (67.2%)	21 (32.8%)	
